# Optimization of corneal preservation media with novel antifungal agents

**DOI:** 10.1007/s00203-026-04848-z

**Published:** 2026-03-28

**Authors:** Paula Reginatto, Giovanna de Jesus Agostinetto, Claudete Inês Locatelli, Felipe Silva Guareze, Angélica Rocha Joaquim, Maria Eduarda Krummenauer, Rúbia do Nascimento Fuentefria, Marilene Henning Vainstein, Diane Ruschel Marinho, Saulo Fernandes de Andrade, Alexandre Meneghello Fuentefria

**Affiliations:** 1 Programa de Pós-Graduação em Ciências Farmacêuticas, Faculdade de Farmácia, Universidade Federal do Rio Grande do Sul, Porto Alegre, Brazil; 2 Faculdade de Farmácia, Universidade Federal do Rio Grande do Sul, Porto Alegre, Brazil; 3 Serviço de Oftalmologia, Hospital de Clínicas de Porto Alegre, Porto Alegre, Brazil; 4 Faculdade de Farmácia, Universidade Federal de Santa Maria, Santa Maria, Brazil; 5 Centro de Biotecnologia, Universidade Federal do Rio Grande do Sul, Porto Alegre, Brazil; 6 Hospital de Clínicas de Porto Alegre, Porto Alegre, Brazil

**Keywords:** Cornea, Antifungal, Transplant, Biofilm, *Fusarium*, *Candida*

## Abstract

The performance of ophthalmic surgeries, particularly penetrating keratoplasty, is associated with an increased risk of ocular infections, especially fungal keratitis. In addition to infections such as post-surgical occlusions, microbial contamination of the donor’s tissue, predominantly fungal in nature, can also lead to the disposal of said tissue. The genus *Fusarium*, along with several species from the *Candida* genus, emerge as the primary fungal agents, displaying notable biofilm-forming abilities on surfaces. To address these issues, the addition of antifungals to preservation media vials has been explored, although conventional antifungal agents exhibit limitations. In light of this, compounds such derived from 8-hydroxyquinoline, clioquinol (CLQ) and PH151, emerge as promising alternatives. Hence, this study aimed to assess the efficacy of two derivatives of 8-hydroxyquinoline, both individually and in combination with antifungal agents, as additives in preservation media for human corneas intended for transplantation. Given the antifungal potential of CLQ and the combination voriconazole-amphotericin B-clioquinol, the ability to inhibit fungal growth and biofilm formation in human corneas preserved in Optisol-GS medium was investigated, as well as the endothelial viability of this tissue in the presence of these compounds. Among the tested antifungal agents, the combination of voriconazole, amphotericin B, and clioquinol stood out for its excellent antibiofilm action and potential to reduce the fungal load on the surface of the corneas, did not present endothelial toxicity. Thus, the results underscore the promising nature of this combination as a potential additive in hypothermic corneal preservation media, outlining a viable pathway to enhance corneal transplant efficacy.

**Supplementary Information:**

The online version contains supplementary material available at 10.1007/s00203-026-04848-z.

## Introduction

Fungal keratitis represents an infectious disease of significant severity potential, carrying substantial adverse consequences for patients, including vision loss. Remarkably, ocular fungal infections have shown considerable escalation over recent decades, raising substantial concerns in terms of societal and economic impact (Reginatto et al. 2023; Sitnova and Svetozarskiy 2023). Annually, 1.5 to 2 million individuals encounter visual impairment due to corneal pathologies, among which fungal keratitis stands out. The progression of fungal keratitis encompasses lesions induced directly by infectious agents and, indirectly, by swiftly developing inflammatory responses. Corneal fungal infection is also more likely to lead to perforation when compared to other etiologies of keratitis (Sitnova and Svetozarskiy 2023). Specifically, the genus *Fusarium* holds a prominent position among causal agents, contributing to around 40% of cases, while the *Candida* genus is prominent among yeast-like fungi, accounting for approximately 4.5% of cases, although this proportion may vary geographically (Kaur and Kakkar 2010; Sitnova and Svetozarskiy 2023). Documented cases showed an incidence of 61.9% for *Fusarium* spp. among filamentous fungi and 72.22% for *Candida* spp. among yeast-like fungi (Sitnova and Svetozarskiy 2023). Several fungal species within these genera are known to be proficient in forming biofilms, a phenomenon that perpetuates microbial dynamics and provides protection against external assaults, including immune responses and antimicrobial agents (Calvillo-Medina et al. 2019; Sitnova and Svetozarskiy 2023). Such biofilms directly influence the effectiveness of antifungal agents, elevating minimal inhibitory concentrations (MICs), and exacerbating therapeutic challenges in fungal agent eradication (Mukherjee et al. 2012; Schrecker et al. 2021; Ranjith et al. 2022; Sitnova and Svetozarskiy 2023).

Ophthalmic surgical procedures, notably penetrating keratoplasty, emerge as significant risk factors for ocular infections. Particularly, we have fungal keratitis due to the susceptibility conferred by the breach of corneal integrity and the predisposition associated with tissue injury (Reginatto et al. 2023; Sitnova and Svetozarskiy 2023). Globally, over 12 million individuals await corneal transplants; however, suitable tissue availability is substantially restricted, with approximately one cornea for every 70 needy patients (Gain et al. 2016). The COVID-19 pandemic has further exacerbated this shortage. Moreover, a considerable portion of corneal tissue is discarded annually due to microbiological concerns, including fungal contaminations, as well as positive serological results or tissue expiration (Gain et al. 2016; Chaurasia et al. 2020; Rodella et al. 2022). Preventive measures, such as rigorous surveillance of tissue processing and storage, are pivotal (Davila and Mian 2016). Hypothermic preservation in solutions like Optisol-GS, capable of maintaining corneal morphology and thickness, is widely employed (Faria e Sousa and Barretto 2017). However, efforts to combat microbiological contaminations often overlook the presence of antifungals, necessitating antifungal additives to these solutions (Pereira 2011; Krajina et al. 2022).

Nevertheless, the administration of ophthalmic antifungals, such as polyenes and azoles, faces significant challenges, as these compounds frequently exhibit ocular toxicities (Lakhani et al. 2019; Abbondante et al. 2023; Reginatto et al. 2023). Moreover, *Fusarium* spp. strains often display resistance to various therapeutic options, including natamycin and voriconazole (Schrecker et al. 2021). In response to these obstacles, research focuses on the development of broad-spectrum antifungal compounds capable of combating biofilms (Nett 2014; Lakhani et al. 2019). In this context, derivatives of 8-hydroxyquinoline such as clioquinol and PH151 gain prominence, demonstrating potential in prior assays (Pippi et al. 2017; Joaquim et al. 2019; Reginatto et al. 2024, 2025).

Given the aforementioned issue, considering the severity and intricacy that fungal infections and contaminations can pose to corneal tissues intended for transplantation, the need for a preservation medium with broad-spectrum antimicrobial properties (including fungal pathogens), low tissue toxicity, and non-irritating to ocular mucosa is evident. Thus, this study aimed to assess the potential of 8-hydroxyquinoline derivatives and their combinations with antifungal drugs as potential additives in preservation media for human corneas intended for transplantation. This was undertaken to investigate their antifungal and antibiofilm actions, as well as their ability to maintain corneal tissue integrity.

## Materials and methods

### Fungal Strains

We selected 11 strains of the genus *Fusarium*: *F. solani* (ATCC 36031; F28; F33; HCF41), *F. oxysporum* (HCF46; F24; F35), *F. falciforme* (F9), *F. keratoplasticum* (F21; HCF17) and *Fusarium* sp. (SP) and 16 strains of the genus *Candida* were selected: *C. albicans* (CA MT02; CA MT05; CA MT09; CA MT10), *C. tropicalis* (ATCC 750; CT MT16; CT MT23; CT MT28), *C. parapsilosis* (CP MT03; CP MT06; CP MT19; CP MT34) and *C. glabrata* (CG MT01; CG MT12; CG MT17; CG MT29). The fungi belong to the collection of the Research Laboratory in Applied Mycology at the Universidade Federal do Rio Grande do Sul (Porto Alegre, Brazil). The standard strain was obtained from ATCC (American Type Culture Collection, Manassas, VA, USA).

### Compound and antifungal agents

Four antifungal agents were used: natamycin (NAT - Sigma-Aldrich) amphotericin B (AMB – INTERLAB), voriconazole (VRC - Pfizer), clioquinol (CLQ - Sigma-Aldrich) and a novel compound derived from 8-hydroxyquinoline, PH151 (synthesized by the “Pharmaceutical Synthesis Group”). Stock solutions were prepared in DMSO (Sigma-Aldrich) so that when diluted in assay medium, a maximum DMSO concentration of 2% was obtained. The minimum inhibitory concentrations of antifungal agents and combinations come from previous studies, according to the information Table 1 (Reginatto et al. 2024; Reginatto et al. 2025).

**Table 1:** Minimum Inhibitory Concentration (MIC, µg/m L and µM) of antifungal agents alone and in combination against fungal strains of the genera *Fusarium* and *Candida*

Strains	MIC individual μg/mL (μM)					MIC combination μg/mL (μM)					
						VRC-AMB-CLQ			VRC-AMB-PH151		
	VOR	NAT	AMB	CLQ	PH151	VRC	AMB	CLQ	VRC	AMB	PH151
CA MT02	512.0 (1,465.74)	32.0 (48.07)	0.5 (0.54)	0.5 (1.64)	2.0 (5.97)	16.0 (45.80)	0.031 (0.034)	0.25 (0.818)	128.0 (366.43)	0.031 (0.034)	0.25 (0.747)
CA MT09	1.0 (2.86)	32.0 (48.07)	0.5 (0.54)	0.5 (1.64)	4.0 (11.95)	0.25 (0.716)	0.125 (0.135)	0.125 (0.409)	0.063 (0.179)	0.625 (0.068)	0.5 (1.49)
ATCC 750	128.0 (366.43)	16.0 (24.03)	0.25 (0.27)	0.5 (1.64)	4.0 (11.95)	8.0 (22.90)	0.016 (0.017)	0.25 (0.818)	8.0 (22.90)	0.031 (0.034)	0.125 (0.373)
CT MT28	8.0 (22.90)	16.0 (24.03)	2.0 (2.16)	1.0 (3.27)	2.0 (5.97)	2.0 (5.72)	0.25 (0.271)	0.25 (0.818)	4.0 (11.45)	0.5 (0.541)	0.125 (0.373)
CP MT03	0.25 (0.71)	32.0 (48.07)	1.0 (1.08)	0.5 (1.64)	4.0 (11.95)	0.125 (0.358)	0.063 (0.068)	0.25 (0.818)	0.125 (0.358)	0.5 (0.541)	0.5 (1.49)
CP MT34	0.5 (1.43)	16.0 (24.03)	1.0 (1.08)	0.5 (1.64)	4.0 (11.95)	0.25 (0.716)	0.031 (0.034)	0.0625 (0.205)	0.25 (0.716)	0.5 (0.541)	0.25 (0.747)
CG MT12	1.0 (2.86)	16.0 (24.03)	1.0 (1.08)	0.5 (1.64)	2.0 (5.97)	0.5 (1.43)	0.063 (0.068)	0.25 (0.818)	0.5 (1.43)	0.125 (0.135)	0.125 (0.373)
CG MT29	0.0312 (0.09)	16.0 (24.03)	0.5 (0.54)	0.5 (1.64)	1.0 (2.99)	0.008 (0.022)	0.016 (0.017)	0.5 (1.64)	0.008 (0.022)	0.125 (0.135)	0.25 (0.747)
ATCC 36031	128.0 (366.43)	16.0 (24.03)	4.0 (4.33)	1.0 (3.27)	4.0 (11.95)	32.0 (91.61)	0.25 (0.271)	0.25 (0.818)	32.0 (91.61)	0.5 (0.541)	0.5 (1.49)
HCF46	8.0 (22.90)	16.0 (24.03)	4.0 (4.33)	1.0 (3.27)	4.0 (11.95)	1.0 (2.86)	0.5 (0.541)	0.5 (1.64)	8.0 (22.90)	0.25 (0.271)	0.125 (0.373)
F9	64.0 (183.22)	16.0 (24.03)	2.0 (2.16)	1.0 (3.27)	4.0 (11.95)	16.0 (45.80)	0.25 (0.271)	0.25 (0.818)	32.0 (91.61)	0.25 (0.271)	0.5 (1.49)
F21	128.0 (366.43)	16.0 (24.03)	4.0 (4.33)	1.0 (3.27)	4.0 (11.95)	32.0 (91.61)	0.5 (0.541)	0.5 (1.64)	16.0 (45.80)	1.0 (1.08)	0.5 (1.49)
F28	64.0 (183.22)	16.0 (24.03)	8.0 (8.66)	1.0 (3.27)	4.0 (11.95)	4.0 (11.45)	0.5 (0.541)	0.5 (1.64)	32.0 (91.61)	4.0 (4.33)	0.125 (0.373)

Data reference: Reginatto et al. (2024) e Reginatto et al. (2025)

VRC: voriconazole; NAT: natamycin; AMB: amphotericin B; CLQ: clioquinol; PH151: 8-hydroxy-quinoline derivative

CA: Candida albicans; CT: Candida tropicalis; CP: Candida parapsilosis; CG: Candida glabrata

HCF46: Fusarium oxysporum; F9: Fusarium falciforme; F21: Fusarium keratoplasticum; F28: Fusarium solani

MIC: minimum inhibitory concentration of each component within the combination

Synergistic VCR-AMB-CLQ Combination: CA MT02; CA MT09; ATCC 750; CT MT28; CP MT34; ATCC 36,031; F28; F24; F9

Synergistic VCR-AMB-PH151 Combination: CA MT02; CA MT09; ATCC 750; CG MT12; CG MT29; ATCC 36,031; F9; F21

### Capacity of biofilm formation in polystyrene microtiter plates

The ability of fungal strains to form biofilms was evaluated in 96-well polystyrene microtiter plates using the technique by Ramage et al. (2001) with modifications. *Candida* spp. and *Fusarium* spp. cell cultures were grown in Sabouraud Dextrose Agar (SDA, KASVI). The colonies were resuspended in Soya Tryptone Broth (TSB, KASVI) at a concentration of 1.0–5.0 × 10^6^ Colony Forming Units per mL (CFU/mL). The fungal inoculum was added to the wells of the plates together with RPMI 1640 medium. Biofilm remaining biomass was evaluated according to the technique described by Stepanović et al. (2007), with modifications. The reading was performed in a spectrophotometer (SpectraMax, KASUAKI) at a wavelength of 450 nm. The test was performed in triplicate.

### Inhibition of biofilm formation in human corneoscleral Halos

To evaluate the ability of the tested antifungal agents to inhibit the formation of biofilm on corneal-scleral halos obtained from the Hospital de Clínicas de Porto Alegre, coming as material for disposal of human corneal transplants. The test was performed in accordance with Reginatto et al. (2020) with modifications. The corneoscleral halos were then washed with sterile saline solution, washed with 70% alcohol and washed again with saline solution. Then, they were placed in 24-well plates containing a solution of antifungal agents plus fungal inoculum. The fungal inoculum was prepared in RPMI 1640 medium at a concentration of 1.0–5.0 × 10^6^ CFU/mL. Five strains of the genus *Fusarium* (ATCC 36031; HCF46; F9; F21; F28) and eight strains of the genus *Candida* (CA MT02; CA MT09; ATCC 750; CT MT28; CP MT03; CP MT34; CG MT12; CG MT29). Antifungal agents were tested at Minimal Inhibitory Concentration (MIC), 10 times the MIC (MICx10) and 50 times the MIC (MICx50) (just for the combinations) concentrations. Plates were incubated for 5 days. After, the halos were washed with sterile saline, added to the tube containing sterile saline, and placed in an ultrasound bath (USC-700; UNIQUE, São Paulo, Brazil) for 40 min at a power of 40 kHz for fungal cell detachment. The same procedures were performed for the halos that were exposed to the solution without the presence of an antifungal agent (untreated control). Then, serial dilutions of ten times were made, and were plated on SDA plates. The plates were incubated, subsequently, the CFU/mL and the percentage of inhibition of biofilm formation compared to the untreated control were determined. The test was performed in triplicate.

### Effectiveness test (death curve)

The VRC-AMB-CLQ combination efficacy trial was performed according to Layer et al. (2014) and Dal Pizzol et al. (2021), with modifications. The selection of strains was based on criteria of clinical relevance (*C*. *albicans*) and the use of a reference strain (*Fusarium*). The effectiveness of the combination was evaluated against a strain of *Fusarium* spp. (ATCC 36031) and a strain of *Candida* spp. (CA MT02) using sterile vials with RPMI 1640. Then, the fungal suspensions were diluted to approximately 2.5 × 10^5^ CFU/mL and 1.0 × 10^6^ CFU/mL in RPMI 1640 vials, for CA MT02 and ATCC 36,031 respectively. The vials were divided into five groups as follows: VRC-AMB-CLQ MIC; VRC-AMB-CLQ MICx10 and VRC-AMB-CLQ MICx50 from the MIC values obtained for each strain, RPMI 1640 not supplemented with antifungal active as a positive control (with fungal inoculum) and RPMI 1640 not supplemented with antifungal active as a negative control (without inoculum). The vials were refrigerated from 4 to 8 °C according to the manufacturer’s recommendations. After 2, 5, 7, 10 and 14 days of incubation, serial dilutions of ten times (up to 10^− 5^) were made, and were plated on SDA plates. The CFU/mL and the percentage of inhibition of biofilm formation compared to the untreated control were determined. The test was performed in triplicate. Two-way ANOVA followed by Dunnett’s test was performed to assess the effectiveness of cell reduction of *Candida* spp. and *Fusarium* spp. were exposed to different concentrations of the association added to RPMI 1640 and compared to the control. *P* < 0.05 was considered statistically significant (95% confidence interval).

### Scanning electron microscopy

The analysis of biofilm formation and treatment on the corneal tissue was performed using scanning electron microscopy. Two fungal strains were used: CA MT02 and ATCC 36,031. The selection of strains was based on criteria of clinical relevance (*C*. *albicans*) and the use of a reference strain (*Fusarium*). Biofilm formation on the corneal tissues was performed as described in the **item 2.4**, where the fungal inoculum was incubated together with the cornea and the combination to be tested (VRC-AMB-CLQ at MICx50 concentration) as well as untreated controls, 48–72 h. After incubation to form a biofilm on the tissue, the samples were prepared for analysis. Scanning electron microscopy preparation was performed according to Lopes et al. (2017) with minor modifications. The tissues were examined with the Auriga Zeiss or Zeiss MA10 microscope. Microscopic fields were randomly selected.

### Cell viability of human corneas

Initially, a screening was performed to evaluate the active ingredient in the preservation medium (solubility and possibility of endothelial counting), three concentrations of the VRC-AMB-CLQ combination were tested: MIC (32 − 2–0.5 µg/mL or 91.61–2.16–1.64 µM), MICx10 (320 − 20–5 µg/mL or 916.09–21.64–16.37 µM) and MICx50 (1600 − 100–25 µg/mL or 4,580.44–108.21–81.83 µM). The MICx50 concentration did not allow endothelial counting due to the large precipitation of antifungal agents, the MICx10 concentration allowed cell counting, however, there was slight precipitation and due to the fact that this precipitation could impair the control and visual evaluation of the viability of the medium (color, microbial growth, color change, dispersed particles), the MIC concentration was chosen, which presented excellent solubility, with no signs of particulate matter in the medium. While MIC values may vary across isolates, the use of the highest observed MIC ensures that safety testing encompasses the upper range of therapeutic exposure, thereby strengthening translational applicability. Three pairs of corneas from donors unfit for transplantation due to positive serology were obtained from the Cornea Eye Bank of Hospital de Clínicas de Porto Alegre. Three pairs of corneas were used to test the VRC-AMB-CLQ combination in MIC concentration (reference value from the strain with the highest MIC value obtained). Randomly, by drawing lots, the Optisol-GS bottle with the randomly selected cornea was supplemented with VRC-AMB-CLQ at a concentration of MIC, and the other cornea of the pair was kept without supplementation as a control (Optisol-GS without combination supplementation). Endothelial cell density (ECD) of all corneas was assessed by specular microscopy (Konan KSS-EB10) after 9 days of conservation. ECDs for paired samples within groups were analyzed using the Wilcoxon test and the Mann-Whitney U test to compare ECD variation between the control and Optisol-GS supplemented groups.

## Results

**Fig. 1:**
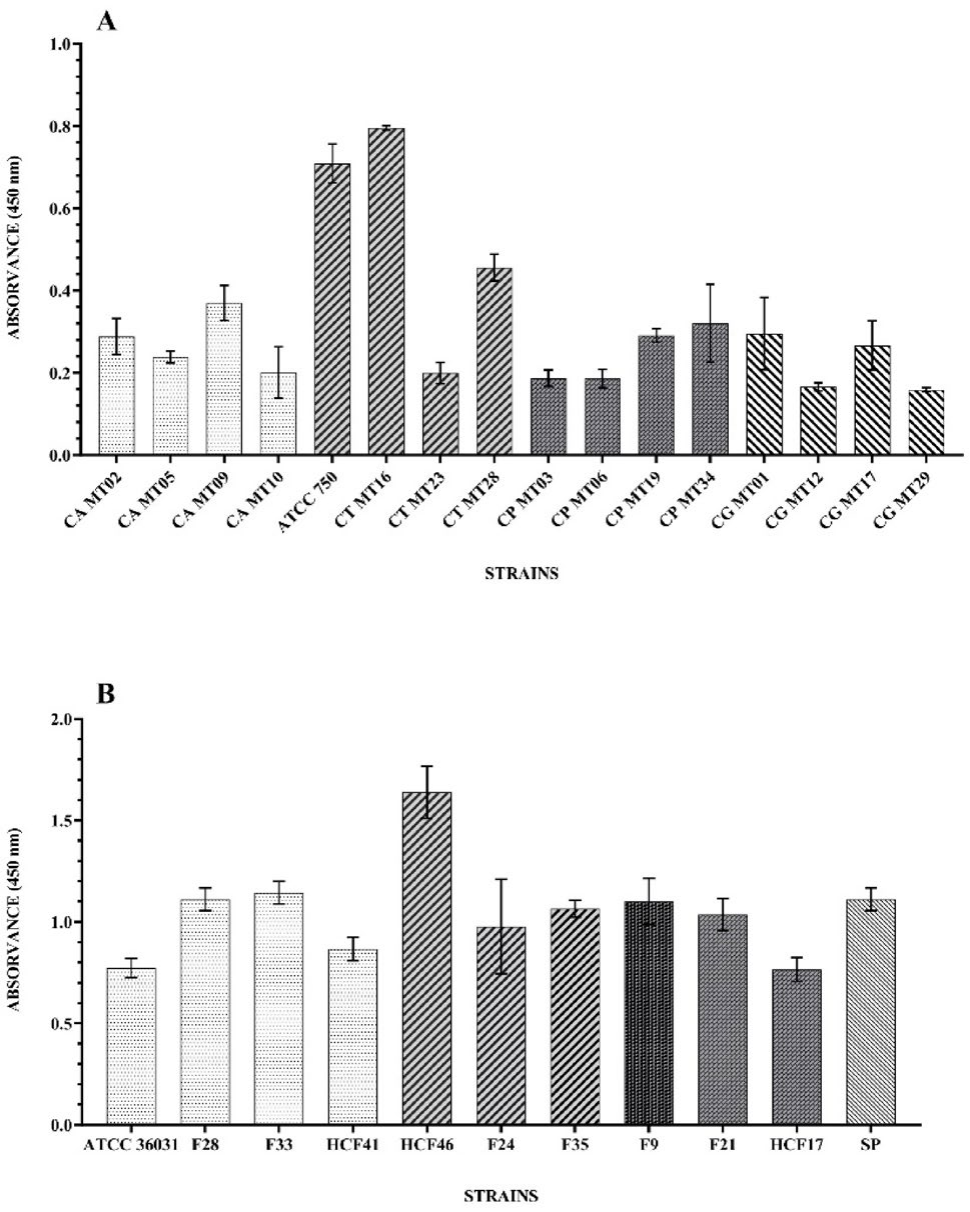
Biofilm formation of *Candida* and *Fusarium* strains in 96-well polystyrene microtiter plates. A: *Candida* strains (*Candida albicans*: CA MT02; CA MT05; CA MT09; CA MT10, *Candida tropicalis*: ATCC 750; CT MT16; CT MT23; CT MT28, *Candida parapsilosis*: CP MT03; CP MT06; CP MT19; CP MT34, *Candida glabrata*: CG MT01; CG MT12; CG MT17; CG MT29). B: *Fusarium* strains (*Fusarium solani*: ATCC 36031; F28; F33; HCF41, *Fusarium oxysporum*: HCF46; F24; F35, *Fusarium falciforme*: F9, *Fusarium keratoplasticum*: F21; HCF17, *Fusarium* sp.: SP)

In the current study, all employed strains were subjected to an assessment of their in vitro biofilm-forming ability using 96-well polystyrene microtiter plates. It was observed that all strains exhibited varying capacities for biofilm formation, as depicted in Fig. 1 (intervals and capacities displayed in the figure). Predominantly, *Fusarium* strains demonstrated optical densities (OD) exceeding 1.0, with the exception of four strains, ATCC 36,031 and HCF41 (*F. solani*), HCF17 (*F. keratoplasticum*), and F24 (*F. oxysporum*). Conversely, *Candida* genus strains predominantly displayed moderate to high biofilm-forming capacities, with the exception of strains CG MT12 and CG MT29, characterized as low-intensity biofilm formers, as their optical densities remained below 0.170.

For the analysis of the inhibitory potential of antifungal agents and their combinations concerning biofilm formation on corneoscleral halos, 13 strains were utilized (eight from the *Candida* spp. genus and five from the *Fusarium* spp. genus). Among *Candida* spp., both antifungal agents and their combinations, tested at MIC and MICx10 concentrations, achieved growth inhibition rates exceeding 90%. Remarkably, certain combinations achieved 100.00% inhibition for strains ATCC 750 and CG MT12, as shown in Table 2. Regarding *Fusarium* genus strains, among the obtained results, inhibitions exceeding 90.00% were also observed. However, particularly at MIC concentrations, for some strains, the found inhibition percentages did not reach 90.00% (Table 2). At the corresponding MICx50 concentration, both combinations were effective in inhibiting fungus adhesion to tissue, showing inhibition rates greater than 96%. Notably, in the case of the VRC-AMB-CLQ combination, the inhibition rates were remarkable, exceeding 99%. There were only two exceptions, 98.95% for the HCF46 strain and 97.45% for the F28 strain.

**Table 2:** Inhibition of biofilm formation (%) of *Candida* and *Fusarium* strains on human corneal-scleral halos following treatment with antifungal agents alone or in combination

Strains	INIBITION (%)															
	VRC		NAT		AMB		CLQ		PH151		VRC-AMB-CLQ			VRC-AMB-PH151		
	MIC	MICx10	MIC	MICx10	MIC	MICx10	MIC	MICx10	MIC	MICx10	MIC	MICx10	MICx50	MIC	MICx10	MICx50
CA MT02	99.70	99.86	96.29	100.00	96.77	100.00	94.85	99.86	76.86	95.91	99.72	99.86	99.93	98.91	99.93	99.93
CA MT09	96.65	96.38	96.17	99.49	95.07	99.51	96.73	98.48	88.47	98.84	96.65	99.75	99.89	95.07	95.07	99.81
ATCC 750	100.00	100.00	100.00	100.00	99.92	99.92	90.12	100.00	84.42	100.00	100.00	100.00	100.00	99.92	99.92	100.00
CT MT28	99.61	99.62	99.34	99.92	90.76	99.93	88.75	100.00	89.24	99.47	99.71	99.71	99.98	90.76	95.63	99.96
CP MT03	76.48	95.79	98.64	99.60	70.85	93.04	78.52	94.09	82.05	86.47	87.70	99.14	99.60	72.94	83.95	99.41
CP MT34	87.37	91.74	93.05	96.38	90.66	95.49	84.99	89.59	83.48	91.74	88.43	94.02	99.82	90.66	93.12	99.82
CG MT12	99.33	100.00	99.83	100.00	100.00	100.00	17.79	98.61	88.99	96.18	99.33	99.75	100.00	99.69	100.00	100.00
CG MT29	90.70	94.44	93.59	99.38	94.20	100.00	91.63	97.58	99.31	99.96	99.29	99.92	100.00	92.69	96.39	99.98
ATCC 36031	92.06	97.90	84.12	97.90	80.42	87.40	95.80	99.86	65.80	95.80	95.80	97.90	100.00	92.06	95.80	97.90
HCF46	97.90	98.95	93.44	97.90	90.21	90.21	97.90	98.95	96.85	97.90	98.95	100.00	98.95	96.85	99.92	99.92
F9	97.97	100.00	98.64	100.00	93.70	96.31	79.72	97.97	93.84	99.32	100.00	100.00	100.00	99.32	100.00	99.32
F21	97.98	99.73	98.63	100.00	70.91	98.84	89.91	97.38	97.51	99.73	100.00	100.00	100.00	98.16	99.46	99.73
F28	93.06	96.18	75.89	96.18	90.38	92.06	90.38	94.49	75.33	93.94	92.36	95.19	97.45	93.47	96.18	96.18

VRC: voriconazole; NAT: natamycin; AMB: amphotericin B; CLQ: clioquinol; PH151: 8-hydroxy-quinoline derivative

CA: Candida albicans; CT: Candida tropicalis; CP: Candida parapsilosis; CG: Candida glabrata

HCF46: Fusarium oxysporum; F9: Fusarium falciforme; F21: Fusarium keratoplasticum; F28: Fusarium solani

MIC: minimum inhibitory concentration of each component within the combination

MICx10: value of the minimum inhibitory concentration of each component within the combination multiplied by 10

MICx50: value of the minimum inhibitory concentration of each component within the combination multiplied by 50

Values ≥ 90% indicate strong inhibition of adherence/biofilm formation

**Fig. 2:**
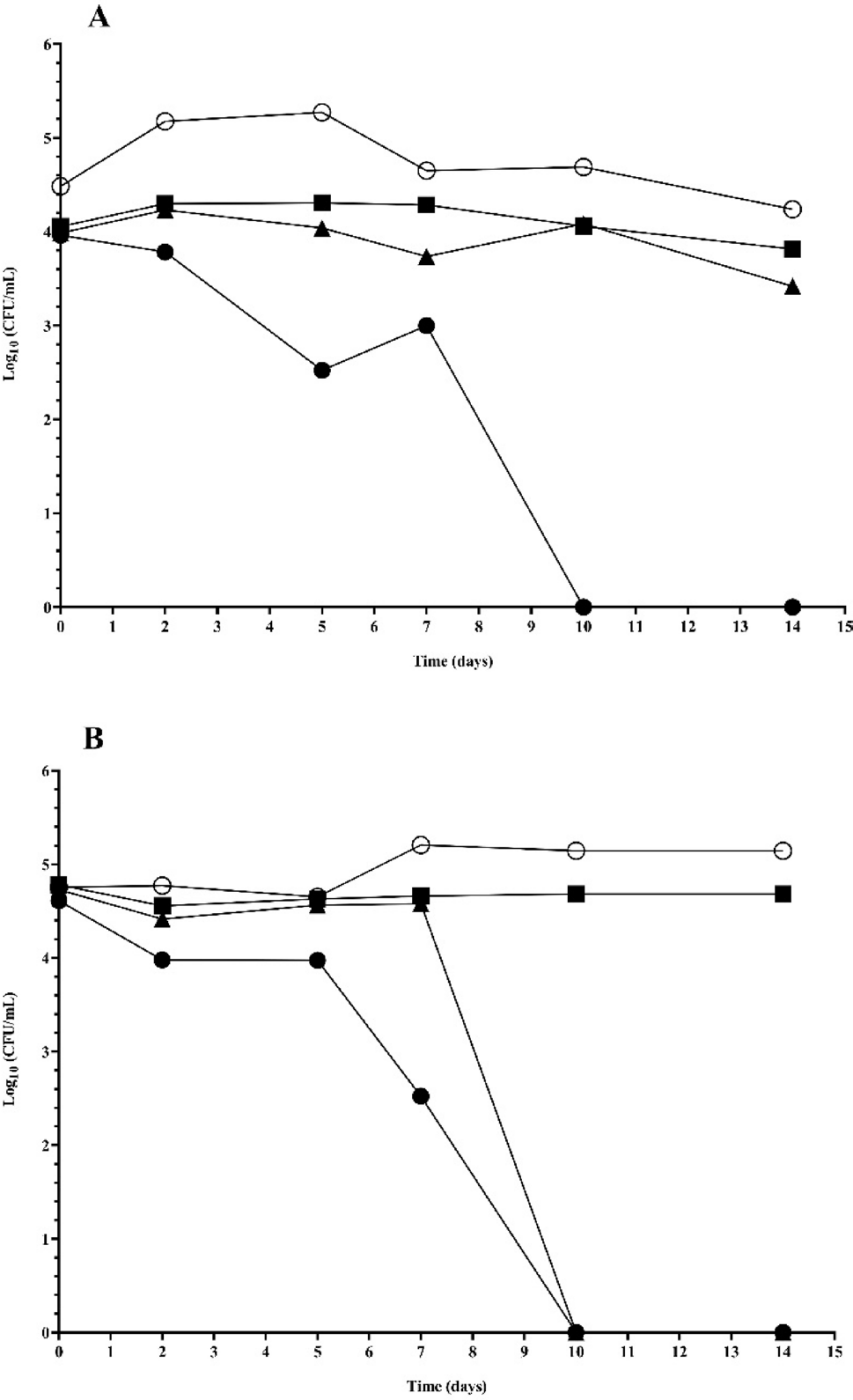
Evaluation of the effectiveness of the combination VRC-AMB-CLQ (voriconazole-amphotericin B-clioquinol) on MIC (■), MICx10 (▲) and MICx50 (●) concentrations over 14 days (times 0, 2, 5, 7, 10 and 14 days) at 4–8 °C against two different strains (growth control ○). A: *Candida albicans* strain: CA MT02. B: *Fusarium solani* strain: ATCC 36,031

The investigation into the efficacy of the selected combination (VRC-AMB-CLQ) at the MIC concentration revealed a growth curve with a slight reduction in fungal counts compared to the control group for both evaluated genera (CA MT02 and ATCC 36031). Even when the MICx10 concentration was employed, the effectiveness of the combination did not exhibit a substantial reduction in strain growth over the assessed time points. However, for the ATCC 36,031 strain, colony counts became undetectable from the 10th day onwards, with cellular counts reduced by over 99.9%, indicating a fungicidal action of the combination at this time and concentration. Upon evaluating the combination at the MICx50 concentration, a pronounced curve of fungal growth reduction was observed, resulting in non-detectable levels in plating analyses (Fig. 2), with reductions exceeding 99.9% from the 10th day for both strains (CA MT02 and ATCC 36031), hence considered to possess fungicidal action.

**Fig. 3:**
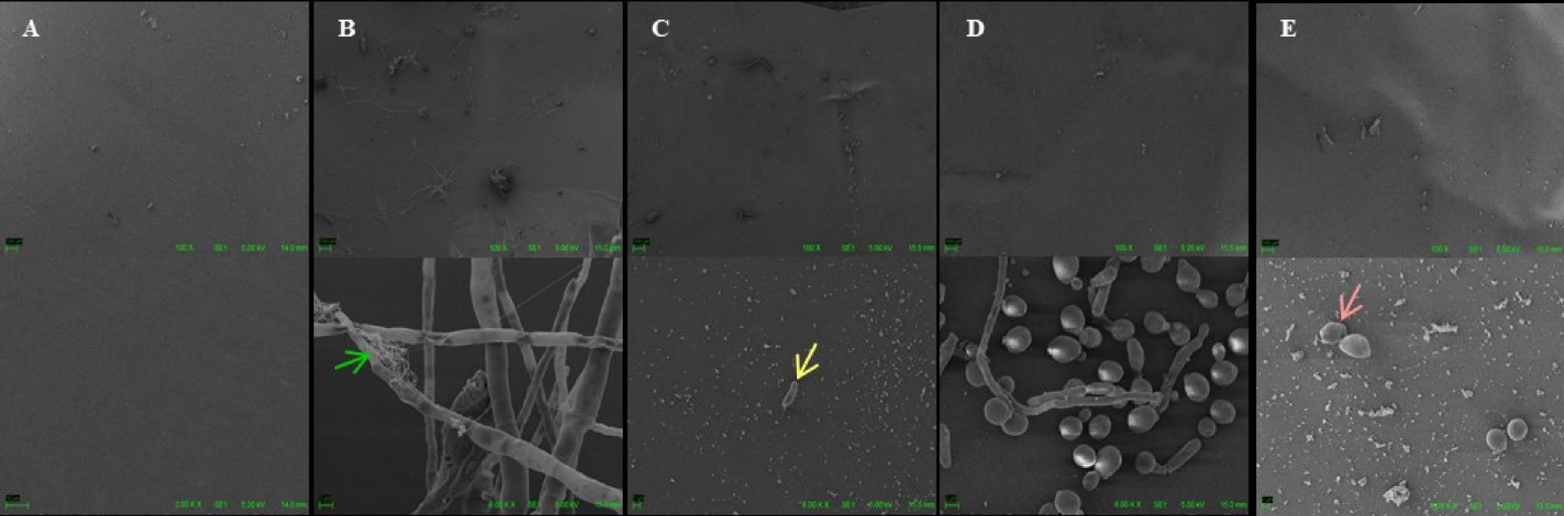
Scanning electron microscopy of corneal tissues with and without treatment against strains ATCC 36,031 and CA MT02: (A) Cornea without treatment and without addition of fungus (100x and 6000x); (B) Cornea without treatment in the presence of strain ATCC 36,031 (100x and 6000x); (C) Cornea treated with VRC-AMB-CLQ MICx50 in the presence of strain ATCC 36,031 (100x and 6000x); (D) Cornea without treatment in the presence of the CA MT02 strain (100x and 6000x); (E) Cornea treated with VRC-AMB-CLQ MICx50 in the presence of CA MT02 strain (100x and 6000x). Green arrow: extracellular matrix; Yellow arrow: conidium; Pink arrow: morphologically misshapen blastoconidium

The strains tested, ATCC 36,031 and CA MT02, showed little growth on corneal tissue (control without treatment), however, enough to observe a very significant reduction in relation to the tissues treated with the combination VRC-AMB-CLQ MICx50, through images obtained by scanning electron microscopy (Fig. 3).

**Fig. 4:**
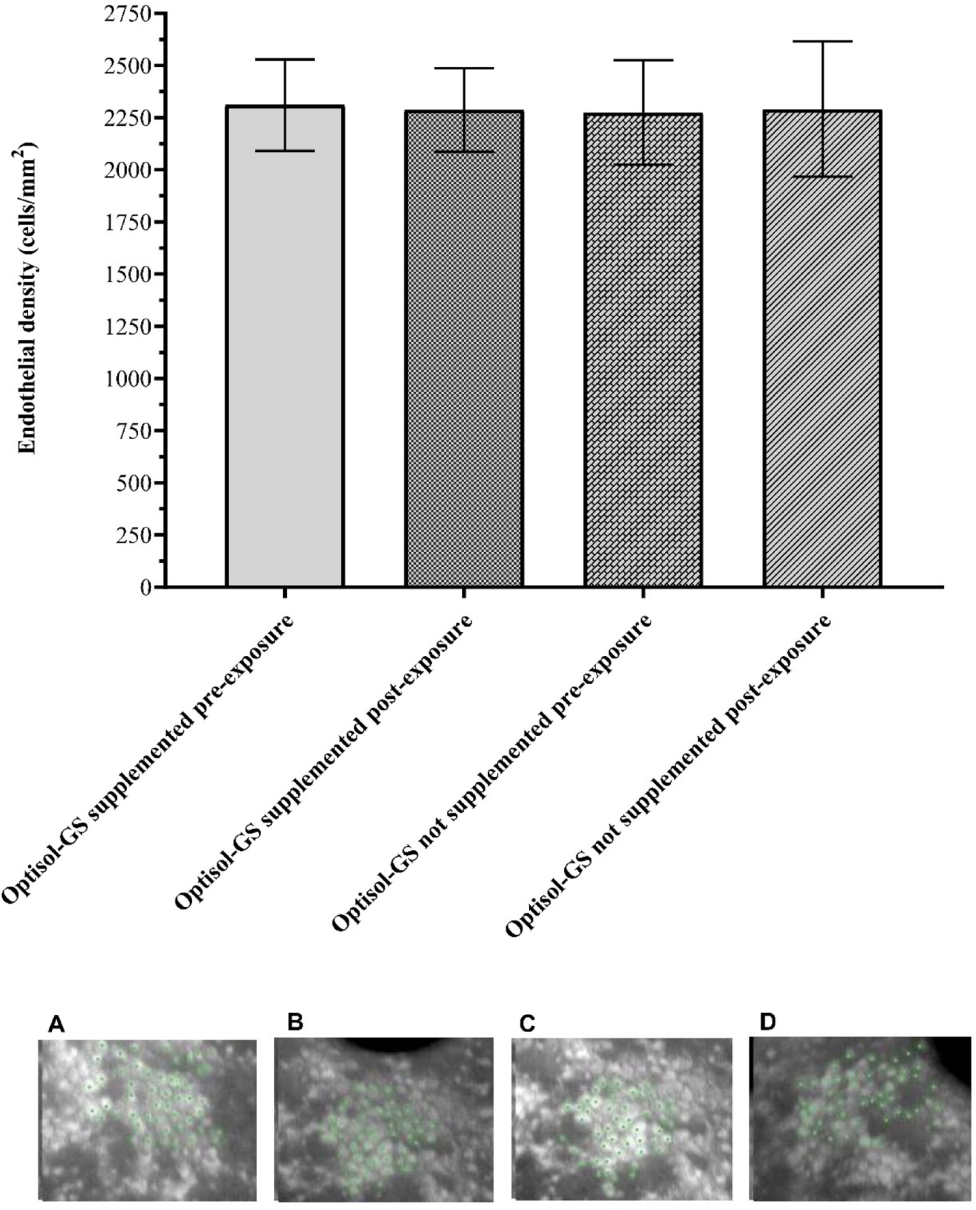
Endothelial density evaluated by specular microscopy of corneal tissue stored in Optisol-GS preservation medium, initial storage of 48 h and after 9 days of exposure to the preservation medium supplemented and not supplemented with the triple combination VRC-AMB-CLQ (voriconazole-amphotericin B-clioquinol). (A) Cornea preserved in Optisol-GS preservation medium to be supplemented with the combination VRC-AMB-CLQ (48 h, pre-exposure); (B) Cornea preserved in Optisol-GS preservation medium supplemented with the combination VRC-AMB-CLQ (9 days, post-exposure); (C) Cornea preserved in Optisol-GS preservation medium not supplemented with antifungal agents (48 h, pre-exposure); (D) Cornea preserved in Optisol-GS preservation medium not supplemented with antifungal agents (9 days, post-exposure)

Corneal tissue storage for 9 days in preservation media, with or without supplementation with the VRC-AMB-CLQ combination, did not result in significant changes in ECDs compared with the baseline endothelial cell count prior to storage (*p* = 0.5 and *p* = 1.0, respectively). No statistically significant difference was observed in the mean variations of endothelial cell density (ECD) before and after storage when comparing the control group (non-supplemented medium) with the group stored in medium supplemented with the combination (*p* = 1.0). Figure 4 shows images of a pair of corneas: one cornea preserved in Optisol-GS supplemented with the combination (VRC-AMB-CLQ) and the other cornea preserved in Optisol-GS without supplementation. The mean ECD in the supplemented group was 2,303 cells/mm² before storage and 2,280 cells/mm² after storage. In the control group, the mean ECD was 2,265 cells/mm² before storage and 2,274 cells/mm² after storage.

## Discussion

Over the past decades, there has been a substantial increase in interest directed towards investigating fungal biofilm formation (Calvillo-Medina et al. 2019; Fritsch et al. 2022; Ranjith et al. 2022; Sitnova and Svetozarskiy 2023). Fungi belonging to the *Fusarium* and *Candida* genera, notorious pathogens in ocular infections, have been the subject of study regarding their potential for biofilm formation in the ophthalmological context (Fritsch et al. 2022). Thus, within this scope, strains from these genera, selected for this study, underwent assessment of their abilities to form biofilms on 96-well polystyrene plates. It was found that all examined strains demonstrated some degree of biofilm-forming capacity, with most *Fusarium* strains exhibiting OD above 1.0. However, four strains stood out for presenting reduced biofilm-forming capabilities, including the ATCC 36,031 strain, whose weak biofilm-forming capacity was previously reported in the works of de Chaves et al. (2020) and Abbondante et al. (2023). Concerning *Candida* genus strains, most of them displayed moderate to strong biofilm-forming abilities, as categorized by Bergamo et al. (2014). Notably, only two *C*. *glabrata* strains (CG MT12 and CG MT29) exhibited a less pronounced biofilm-forming profile, of low intensity. This circumstance likely stems from the species’ unique cellular composition, with the absence of hyphae and pseudohyphae, as biofilms formed by microorganisms of this species generally have reduced thickness and consist of compact layers or clusters of yeast-like cells (Silva et al. 2017).

Biofilm formation constitutes a prominent virulence factor that exerts significant influence on the effectiveness of antifungal agents, as the extracellular polymeric matrix provides a protective shield for microorganisms. This matrix plays a role in physical and chemical protection against external challenges, encompassing chemicals, the immune system, and antifungal drugs. In light of these findings, increased structural robustness of the biofilm extracellular matrix restricts the diffusion of antimicrobial agents into deeper cellular layers (Sitnova and Svetozarskiy 2023; Córdoba et al. 2019).

*Post-mortem* obtained corneas for transplant purposes are susceptible to microbiological contamination, and the predominant nature of such contamination is fungal (62%) (Arbelaez et al. 2015; Davila and Mian 2016; Krajina et al. 2022). Biofilm formation during infection or on donor tissue increases the minimal inhibitory concentrations (MICs) required for antifungal efficacy, thereby further complicating the already complex clinical management of fungal ocular infections and contributing to the discard of otherwise eligible transplant tissues (Reginatto et al. 2020; Schrecker et al. 2021; Sitnova and Svetozarskiy 2023). Therefore, it is crucial that antifungal agents incorporated into tissue preservation media effectively inhibit biofilm formation on the surface of donor corneas. This strategy is essential to prevent tissue discard and reduce the risk of post-surgical complications.

Among the tested antifungal agents and combinations, the VRC-AMB-CLQ combination at the MICx10 concentration deserves notable attention, as it achieved results close to 100.00% inhibition, with the exception of only two strains, CP MT34 and F28, which showed inhibitions close to 95.00%. Considering the substantial fungal inoculum used (1.0 × 10⁶ CFU/mL) and the strong biofilm-forming capacity of the strains, these results are both promising and clinically relevant. In this context, given that ocular fungal infections are influenced by factors such as inoculum size and fungal pathogenicity (Durand 2017; Mills et al. 2020), these findings are of particular importance, especially in light of the rapid progression and severe tissue damage associated with *Fusarium* spp. infections (Czakó et al. 2019; Reginatto et al. 2023). Notably, the biofilm requires higher concentrations (Ranjith et al. 2022; Sitnova and Svetozarskiy 2023), and the combination in increasing concentrations demonstrated greater inhibition efficacy. This becomes evident when analyzing the inhibition percentages, which predominantly exceeded 99%, when the MICx50 concentration of VRC-AMB-CLQ was evaluated.

Although positive results have been obtained using antifungal agents in isolation, especially at MICx10 concentrations, it is imperative to consider the inherent nuances of each individual antifungal. Such is the case with the high costs associated with voriconazole use, as well as the activity spectrum of NAT and AMB in ocular infections (Prajna et al. 2010; Reginatto et al. 2023). Furthermore, the combination of antifungal agents offers important advantages, as it benefits from different mechanisms of action. This approach allows for an expanded spectrum of activity and the minimization of potential toxic effects by enabling the use of lower doses, ultimately translating into both direct and indirect economic benefits (Fuentefria et al. 2017). As a result, ongoing experiments were conducted to evaluate the combination that exhibited discreet superiority compared to others, namely the VRC-AMB-CLQ combination, whose synergy and ocular safety were previously confirmed by in vitro toxicity tests (Layer et al. 2014).

The efficacy assay of the mentioned combination was conducted over a period of 14 days, representing the maximum time a corneal tissue can be preserved in a preservation medium while maintaining its viability for transplantation. Given the significant interaction, multiple comparisons were performed comparing each treatment group to the control at each time point. MICx50 (*p* < 0.0001) significantly reduced fungal counts at all time points, MICx10 showed significant reductions from day 2 onward (*p* < 0.0001), while MIC demonstrated significant reductions at later time points. In this regard, investigations conducted by Layer et al. (2014) and Dal Pizzol et al. (2021) explored other antimicrobial agents, such as voriconazole, amphotericin B, and cycloheximide, at different concentrations, as additives to Optisol-GS corneal preservation media. Even at higher concentrations, such as MICx5 and MICx25, effective eradication of the fungal inoculum in the solution was not observed. Mostly, the tested drugs managed to stabilize growth curves, resulting in a slight reduction compared to the control group (Layer et al. 2014; Dal Pizzol et al. 2021). The action of triple combination was particularly notable against the *Fusarium* genus, since a known causative agent of challenging ocular infections with high potential for damage (Czakó et al. 2019; Reginatto et al. 2023). The combination demonstrated sustained action over the evaluated 14 days, the maximum recommended storage time for corneal tissues to be donated (Pereira 2011). By doing so, we aimed to account for inter-strain variability and minimize the risk of underestimating possible cytotoxic effects.

As observed in the biofilm formation test in microtiter plates and according to data presented in other Works (de Chaves et al. 2020; Abbondante et al. 2023), the ATCC 36,031 strain formed a discreet biofilm on the tissue of the cornea in the absence of the antifungal combination, presence of hyphae and subtle extracellular matrix formation. The CA MT02 strain exhibited only blastoconidia formation and small fragments of structures such as pseudohyphae. Although *C*. *albicans* is typically expected to form a robust biofilm composed of hyphae, blastoconidia, and extracellular matrix, Gómez-Casanova et al. (2021) reported images demonstrating the presence of blastoconidia alone in this species (Gómez-Casanova et al. 2021). Which may be related to assay conditions. When observing the corneal tissues treated with the combination VRC-AMB-CLQ MICx50, there was an important reduction of fungal structures on the tissue, corroborating the results of the other tests carried out in this work. Furthermore, some structures, including blastoconidia of the CA MT02 strain, were observed on the tissue surface but exhibited morphological alterations, along with a complete absence of hyphae or pseudohyphae. These findings may be related to the mechanism of action of CLQ, which inhibits hyphal and pseudohyphal formation in *Candida* species (Pippi et al. 2019; You et al. 2020). The ability to eliminate cells present in the preservation solution is also crucial for mitigating contamination that may lead to donor cornea discard. This is particularly important given the large number of patients awaiting transplantation and the considerable number of corneal tissues discarded annually due, among other factors, to microbial contamination, predominantly of fungal origin (Arbelaez et al. 2015; Davila and Mian 2016; Gain et al. 2016; Rodella et al. 2022).

The commercial preservation medium Optisol-GS, supplemented with the antifungal combination, maintained corneal preservation efficacy and preserved endothelial cell density. In addition, the formulation provided antimicrobial and antifungal activity. Endothelial integrity was preserved in corneas evaluated 9 days after storage, with no significant differences observed between the control group (unsupplemented medium) and the test group (medium supplemented with the antifungal combination). Although the antifungal and antibiofilm efficacy of the VRC–AMB–CLQ combination has been experimentally demonstrated, its clinical application requires compliance with applicable regulatory frameworks. In Brazil, this responsibility falls under the National Health Surveillance Agency (ANVISA), which requires validation of safety, sterility, physicochemical stability, and tissue compatibility for any modifications to ocular preservation media. The addition of non-standardized antifungal combinations would require further regulatory studies and possible revalidation of the product as an approved input for use in eye banks. Large-scale implementation of such an additive in preservation media presents challenges, including inter-batch standardization, quality control, stability during refrigerated storage, and economic impact. Currently, individual antifungals such as amphotericin B and voriconazole are being experimentally explored in eye bank practice, although without definitive regulatory consensus. In contrast, other studies using single antifungal agents as active ingredients required higher concentrations to achieve antifungal efficacy. Although these studies demonstrated important antifungal potential, the safety of such agents on corneal tissue, particularly regarding endothelial viability, remains to be fully established. Reports of toxicity and/or significant reductions in endothelial cell density indicate that these antifungals, at the tested concentrations, warrant greater caution (Ritterband et al. 2007; Layer et al. 2014; Tran et al. 2019; Dal Pizzol et al. 2021). The triple combination used did not interfere with the corneal tissue preservation capacity of the Optisol-GS medium, presenting an excellent endothelial safety profile, maintaining cell counts similar to the unsupplemented commercial medium. Therefore, the use of this combination of antifungal agents demonstrates significant potential as a supplemental strategy in corneal preservation media compared to the use of a single antifungal agent.

## Conclusion

The VRC-AMB-CLQ and VRC-AMB-PH151 combinations demonstrated antibiofilm efficacy against various strains of *Candida* and *Fusarium* in corneo-scleral halo tissues, even when challenged with strains of high biofilm-forming capacity. Additionally, the VRC-AMB-CLQ combination, selected for further testing, exhibited the ability to decrease cell counts over the 14-day evaluation period, with fungicidal action applicable to both fungal genera. The triple combination used did not interfere with the Optisol-GS medium’s ability to preserve corneal tissue, which indicates that it is an promising antifungal active ingredient. The potential use of this combination as an additive in corneal preservation media holds significant relevance for public health. It represents a promising alternative for reducing the risk of infectious complications following corneal transplantation. Furthermore, this approach assumes a crucial role as a strategy to alleviate the loss of donor tissue, which is eligible for transplantation but is currently discarded due to the presence of microbiological contamination. The relatively limited sample size may have reduced the statistical power to detect subtle differences between groups and may restrict the generalizability of the findings. Furthermore, the absence of standardized, large-scale validation protocols limits the extrapolation of these results to routine eye bank operations. Future studies with expanded sample sizes and harmonized methodological frameworks are necessary to ensure reproducibility and support safe process scale-up. Despite the translational potential, clinical implementation depends on complementary studies and specific sanitary approval.

## Research ethics committee

Samples of human tissue (corneas and corneal-scleral halos) were obtained from the Cornea Eye Bank of the Hospital de Clínicas in Porto Alegre. All donor corneas were donated voluntarily, with written informed consent, and the study methodology followed the guidelines of the Declaration of Helsinki. Ethics committee approval and institutional research board clearance were obtained from the Universidade Federal do Rio Grande do Sul and Hospital de Clínicas de Porto Alegre, registered on the Platforma Brazil, opinion number: 4,283,197. The study was conducted under a waiver of the Free and Informed Consent Form approved by the institutional ethics committee, Consent to Participate declarations: not applicable (Supplementary Material).

## Supplementary Information

Below is the link to the electronic supplementary material.

Supplementary Material 1

## Data Availability

No datasets were generated or analysed during the current study.
