# Supplementary material for: Optimization of corneal preservation media with novel antifungal agents
Source: Arch Microbiol. 2026 Mar 28;208(6):294. doi: 10.1007/s00203-026-04848-z (PMC13032951; doi:10.1007/s00203-026-04848-z)
Supplement: Supplementary file 1 — Supplementary Material 1 [file 203_2026_4848_MOESM1_ESM.pdf]

SUPPLEMENTARY MATERIAL

OPTIMIZATION OF CORNEAL PRESERVATION MEDIA WITH NOVEL ANTIFUNGAL AGENTS

Paula Reginatto[a;b*]; Giovanna de Jesus Agostinetto[a,b]; Claudete Inês Locatelli[c]; Felipe Silva Guareze[c]; Angélica Rocha Joaquim[d]; Maria Eduarda Krummenauer[e]; Rúbia do Nascimento Fuentefria[f]; Marilene Henning Vanstein[e]; Diane Ruschel Marinho[c]; Saulo Fernandes de Andrade[a;b]; *Alexandre Meneghello Fuentefria*[a;b]

[a]Programa de Pós-Graduação em Ciências Farmacêuticas, Faculdade de Farmácia, Universidade Federal do Rio Grande do Sul, Porto Alegre, Brazil.

[b]Faculdade de Farmácia, Universidade Federal do Rio Grande do Sul, Porto Alegre, Brazil.

[c]Serviço de Oftalmologia, Hospital de Clínicas de Porto Alegre, Porto Alegre, Brazil.

[d]Faculdade de Farmácia, Universidade Federal de Santa Maria, Santa Maria, Brazil.

[e]Centro de Biotecnologia, Universidade Federal do Rio Grande do Sul, Porto Alegre, Brazil.

[f]Hospital de Clínicas de Porto Alegre, Porto Alegre, Brazil.

PARECER CONSUBSTANCIADO DO CEP

DADOS DO PROJETO DE PESQUISA

Título da Pesquisa: DESENVOLVIMENTO DE NOVO MEIO DE PRESERVAÇÃO DE CÓRNEAS HUMANAS PARA TRANSPLANTE COM ESPECTRO DE AÇÃO ANTIFÚNGICO

Pesquisador: Alexandre Meneghello Fuentefria

Área Temática:

Versão: 3

CAAE: 28474820.5.0000.5347

Instituição Proponente: Faculdade de Farmácia

Patrocinador Principal: Financiamento Próprio

DADOS DO PARECER

Número do Parecer: 4.283.197

Apresentação do Projeto:

Trata-se do projeto de pesquisa que tem como pesquisador responsável Alexandre Meneghello Fuentefria, intitulado "DESENVOLVIMENTO DE NOVO MEIO DE PRESERVAÇÃO DE CÓRNEAS HUMANAS PARA TRANSPLANTE COM ESPECTRO DE AÇÃO ANTIFÚNGICO" a ser executado de 03/2020 a 03/2022 e que pretende “avaliar a segurança do novo meio de preservação desenvolvido sobre o tecido da córnea.

Como hipótese, os pesquisadores informam que “o desenvolvimento de um novo meio de preservação de córneas alternativo, totalmente nacional, contendo um agente antifúngico inédito irá controlar o crescimento oportunista de fungos patogênicos nesses meios, os quais geralmente são os principais causadores de ceratite e endoftalmite pós-transplante.”.

Foi apresentada uma fundamentação teórica bem estruturada, considerando aspectos relativos às infecções fúngicas, oculares, fatores de risco e tratamentos.

Trata-se de um projeto que será avaliado pelo CEP/UFRGS (Instituição Proponente) referente a projeto de tese de doutorado junto ao Programa de Pós-Graduação em Ciências Farmacêuticas, Faculdade de Farmácia, UFRGS.

Objetivo da Pesquisa:

Como objetivo geral: desenvolvimento de um novo meio de preservação de córneas alternativo, totalmente nacional, contendo um agente antifúngico inédito para o controle de fungos potencialmente resistentes causadores de ceratite e endoftalmite pós-transplante.

Como objetivos específicos:

a) Determinar o perfil de suscetibilidade das cepas fúngicas selecionadas (Fusarium solani, F. oxysporum, F. verticillioides, Candida albicans, C. tropicalis, C. parapsilosis, C. glabrata e Aspergillus fumigatus) frente aos antifúngicos de escolha no tratamento da ceratite e endoftalmite;

b) Caracterizar as cepas quanto a sua capacidade de formação de biofilme antes e depois do cultivo no meio de preservação (Optisol-GS®);

c) Síntese de polímeros para formação de sistemas de liberação controlada específica e seletiva de antifúngicos (para adição no meio de preservação);

d) Desenvolvimento farmacotécnico de um novo meio de preservação de córneas contendo o antifúngico PH151;

e) Avaliação da atividade antifúngica do novo meio de preservação de córneas contendo o antifúngico PH151 sobre fungos patogênicos sensíveis e resistentes aos antifúngicos de escolha no tratamento da ceratite e endoftalmite;

f) Avaliar a atividade antibiofilme do novo meio de preservação de córneas contendo o antifúngico PH151 em córneas estéreis cedidas pelo Serviço de Oftalmologia do HCPA;

g) Avaliar a eficácia antifúngica do novo meio de preservação sobre a córnea infectada com diferentes espécies de fungos, inclusive multirresistentes;

h) Avaliação da morfologia endotelial da córnea no novo meio de preservação com a finalidade de esclarecer a segurança toxicológica no tempo de conservação;

i) Avaliação complementar da segurança do novo meio de preservação desenvolvido sobre o tecido da córnea.

Avaliação dos Riscos e Benefícios:

Na nova versão, foram apresentados como riscos a potencial quebra de confidencialidade e as medidas para minimiza-la (PENDÊNCIA ATENDIDA)

Como benefícios, os pesquisadores relatam o “desenvolvimento de um novo meio de preservação de córneas alternativo aos já existentes, mais barato, totalmente nacional, com espectro de ação

antimicrobiano mais amplo (abrangendo fungos também, inclusive multirresistentes) e seguro. Assim, espera-se alcançar resultados promissores para a prática clínica, com produção de tecnologias e produtos de propriedade intelectual de grande importância em saúde pública. Ainda, enriquecer e aprofundar conhecimentos com publicações de alto impacto e a submissão de uma patente com o novo meio de preservação de córneas."

Comentários e Considerações sobre a Pesquisa:

Conforme informado pelos pesquisadores, "os experimentos e avaliações microbiológicas serão realizados nas dependências do Laboratório de Micologia Aplicada, localizado no anexo II da Faculdade de Farmácia da UFRGS. Os ensaios de síntese serão realizados na mesma Instituição, no Laboratório do "Pharmaceutical Synthesis Group". A avaliação da morfologia endotelial da córnea no novo meio de preservação com a finalidade de esclarecer a segurança no tempo de conservação (ensaios toxicológicos) será realizado no serviço de Oftalmologia do HCPA."

Na nova versão anterior foi incluído Termo de Concordância assinado pela pesquisadora Diane Marinho, identificada como responsável pelo setor de oftalmologia do HCPA (PENDÊNCIA ATENDIDA).

Serão realizados ensaios preliminares em cepas fúngicas a partir da coleção de fungos do laboratório do Grupo de Pesquisa em Micologia Aplicada da UFRGS. Posteriormente será realizado ensaio de síntese de novo antifúngico que será acrescido ao meio de preservação. Novamente as cepas padrão serão testadas neste novo composto formado.

A partir deste ponto, será realizada avaliação da atividade antifúngica sobre a córnea infectada. Conforme descrito pelo pesquisador "Todos os halos córneos-esclerais preservados em Optisol-GS® (Bausch+Lomb) utilizados em transplantes de córnea no HCPA serão divididos após a cirurgia no bloco cirúrgico: uma das metades será colocada no tubo com o meio comercial e a outra metade será colocada no tubo contendo o novo meio de preservação. Os tubos serão refrigerados a 4°C por 24h. Após esse período, os meios receberão uma alíquota de inóculo fúngico (concentrações testadas para leveduras 1-5 x 105 UFC/mL e para fungos filamentosos 1-3 x 104 UFC/mL). Nos tempos 8, 12, 24, 36 e 48 horas de incubação, alíquotas de 10 uL serão removidas e plaqueadas em ágar sabouroud dextrose, incubação a 35 ºC e posterior determinação do número de UFC."

Após, a partir deste material, será realizada a aferição da capacidade de prevenção da formação do biofilme fúngico sobre a córnea.

Posteriormente, será realizado ensaio da membrana cório-alantoide de ovo embrionado de galinha ou HET-CAM.

Ao final do projeto são citados os números amostrais necessários para cada experimento. Conforme citado pelo pesquisador, “Um total de aproximadamente 117 amostras (o que contempla o botão córneo-escleral como um todo ou apenas o halo deste botão), como possíveis erros nos procedimentos dos ensaios poderão ocorrer, dessa forma, o n final total será de 129 amostras (cerca de 10% a mais sobre o número total necessário).” Na Plataforma Brasil é citado 36 indivíduos. Esclarecer se cada indivíduo fornecerá mais de um tecido ou de que forma será compatibilizado o tamanho amostral. Também há necessidade de justificativa para tamanho amostral. Em relação a este ponto, os pesquisadores esclarecem que "Cada ensaio necessita ser realizado, no mínimo, em triplicata para fins de análises estatísticas, controles positivos e negativos também são necessários para fins de comparação e garantia da confiabilidade dos resultados obtidos. Dessa forma, conforme descrito no projeto (compilado abaixo), 129 amostras serão necessárias (halos e botões córneos-esclerais). O número de indivíduos foi uma estimativa muito baixa, na verdade é muito difícil definir o número exato de indivíduos, uma vez que as amostras serão repassadas conforme disponibilidade e viabilidade das mesmas para o estudo. Assim, cada indivíduo pode doar as duas córneas ou apenas uma.

Considerando a justificativa anterior, o projeto de pesquisa deverá informar que se trata de amostra de conveniência. Se serão necessárias 129 amostras e, eventualmente, cada doador pode doar apenas uma córnea, este número deverá ser informado como tamanho amostral. Na nova versão da PB o número amostral foi alterado para 129. (PENDÊNCIA ATENDIDA)

Em relação ao financiamento, foi destacado que a responsabilidade é exclusiva do pesquisador principal. (PENDÊNCIA ATENDIDA).

Em função do parecer anterior ter sido emitido em 02/2020 e o retorno ter ocorrido somente em 09/2020, o cronograma foi adequado para início em 10/2020. (PENDÊNCIA ATENDIDA)

Considerações sobre os Termos de apresentação obrigatória:

Apresentados.

Foi solicitada dispensa do TCLE com a seguinte justificativa: O projeto utilizará halos córneos-esclerais que seriam descartados após o procedimento de transplante.

Conclusões ou Pendências e Lista de Inadequações:

O projeto de pesquisa encontra-se em condições de aprovação, de acordo com os aspectos éticos (CNS Resolução 466/12).

Considerações Finais a critério do CEP:

APROVADO.

Este parecer foi elaborado baseado nos documentos abaixo relacionados:

Tipo Documento	Arquivo	Postagem	Autor	Situação
Informações Básicas do Projeto	PB_INFORMAÇÕES_BÁSICAS_DO_PROJETO_1501404.pdf	13/09/2020 00:52:12		Aceito
Outros	Oficio_HCPA.pdf	13/09/2020 00:49:38	Paula Reginatto	Aceito
Outros	Carta_Resposta_CEP_Parecer.pdf	13/09/2020 00:41:47	Paula Reginatto	Aceito
Projeto Detalhado / Brochura Investigador	Projeto_Paula_Reginatto_CEP_Corrigido.pdf	13/09/2020 00:38:56	Paula Reginatto	Aceito
Declaração de concordância	Oficio_PSG.pdf	07/02/2020 22:59:51	Paula Reginatto	Aceito
Folha de Rosto	Folha_de_Rosto.pdf	28/01/2020 16:33:15	Alexandre Meneghello Fuentefria	Aceito

Situação do Parecer:

Aprovado

Necessita Apreciação da CONEP:

Não

Endereço: Av. Paulo Gama, 110 - Sala 311 do Prédio Anexo 1 da Reitoria - Campus Centro
Bairro: Farroupilha **CEP:** 90.040-060
UF: RS **Município:** PORTO ALEGRE
Telefone: (51)3308-3738 **Fax:** (51)3308-4085 **E-mail:** etica@propesq.ufrgs.br

PORTO ALEGRE, 17 de Setembro de 2020

Assinado por:
MARIA DA GRAÇA CORSO DA MOTTA
(Coordenador(a))

Endereço: Av. Paulo Gama, 110 - Sala 311 do Prédio Anexo 1 da Reitoria - Campus Centro
Bairro: Farroupilha **CEP:** 90.040-060
UF: RS **Município:** PORTO ALEGRE
Telefone: (51)3308-3738 **Fax:** (51)3308-4085 **E-mail:** etica@propesq.ufrgs.br
